# Overview of Traumatic Brain Injury: An Immunological Context

**DOI:** 10.3390/brainsci7010011

**Published:** 2017-01-23

**Authors:** Damir Nizamutdinov, Lee A. Shapiro

**Affiliations:** 1 Department of Surgery, Texas A & M University Health Science Center, College of Medicine, Temple, TX 76504, USA; dnizamutdinov@medicine.tamhsc.edu; 2 Department of Neurosurgery, Neuroscience Research Institute, Baylor Scott & White Health, Temple, TX 76504, USA

**Keywords:** traumatic brain injury, neuroimmunity, neuroinflammation

## Abstract

Traumatic brain injury (TBI) afflicts people of all ages and genders, and the severity of injury ranges from concussion/mild TBI to severe TBI. Across all spectrums, TBI has wide-ranging, and variable symptomology and outcomes. Treatment options are lacking for the early neuropathology associated with TBIs and for the chronic neuropathological and neurobehavioral deficits. Inflammation and neuroinflammation appear to be major mediators of TBI outcomes. These systems are being intensively studies using animal models and human translational studies, in the hopes of understanding the mechanisms of TBI, and developing therapeutic strategies to improve the outcomes of the millions of people impacted by TBIs each year. This manuscript provides an overview of the epidemiology and outcomes of TBI, and presents data obtained from animal and human studies focusing on an inflammatory and immunological context. Such a context is timely, as recent studies blur the traditional understanding of an “immune-privileged” central nervous system. In presenting the evidence for specific, adaptive immune response after TBI, it is hoped that future studies will be interpreted using a broader perspective that includes the contributions of the peripheral immune system, to central nervous system disorders, notably TBI and post-traumatic syndromes.

## 1. Types of Traumatic Brain Injuries in Humans

### 1.1. Epidemiology of TBI in the United States

A traumatic brain injury (TBI) is an injury that disrupts the normal function of the brain and can be caused by a bump, blow or jolt to the head, rapid acceleration and deceleration of the calvarium, or a penetrating head injury [1]. In 2010, the Centers for Disease Control and Prevention estimated that TBIs accounted for approximately 2.5 million emergency department (ED) visits in the United States. Of these, approximately 87% (2,213,826) were treated and released, 11% (283,630) were hospitalized and discharged, and approximately 2% (52,844) died [2]. The leading causes of non-fatal TBI in the U.S. are falls (35%), motor vehicle-associated accidents (17%) and strikes or blows to the head from/against objects, including sport injuries (17%) [3]. The leading causes of TBI-related deaths are motor vehicle crashes, suicides and falls. In the United States, children aged 0–4 years, adolescents aged 15–19 years, and older adults aged >75 years have the highest rates of TBI-related hospitalizations and deaths among all age groups [3]. Approximately 145,000 children/adolescent (aged 0–19 years) and 775,000 older adults (>75 years) are estimated to be living with substantial and long-lasting limitations in social, behavioral, physical and/or cognitive functioning following a TBI [4]. In every age group, TBI-related ED visit rates are higher for males than for females, which were 800.4 vs. 633.7 cases per 100,000, respectively [2]. Males aged 0–4 years have the highest rates for TBI-related emergency department visits, hospitalizations and deaths combined. Regarding the military, Department of Defense data revealed that from 2000–2011, 235,046 service members (4.2% of the 5,603,720 who served in the Army, Air Force, Navy and Marine Corps) were diagnosed with a TBI [5]. Thus, TBI afflicts millions of people each year, including civilian and military populations. It is pertinent to note that these statistics do not account for those people suffering from concussion/mild TBI who did not receive medical care or had outpatient/office-based visits, estimated by some to be hundreds of thousands, if not millions of people each year [3].

### 1.2. Classification of TBI

The severity of TBIs is typically categorized using the Glasgow Coma Scale and can range from: (a) mild; (b) moderate; to (c) severe [6]. TBI outcomes are often determined by using the Glasgow Outcome Scale, which categorizes gross neurobehavioral ranges of recovery: (a) dead; (b) vegetative state; (c) severe disability; (d) moderate disability; (e) good recovery [7]. An alternative prognosis, using Russell and Smith’s classification, is divided as severe or very severe [8]. Considering that detailed classification helps to determine the severity of injury, informs treatment options and is used to assess prognosis and functional recovery, recent suggestions have indicated that better diagnostic and assessment criteria are needed in the TBI field [9,10].

### 1.3. TBI Prognosis

The effects of TBI can adversely affect quality of life, including cognitive, behavioral, emotional and physical deficiencies. Any one or more of these can negatively impact interpersonal, social and occupational functioning, as well as families, communities and the economy in general [11,12]. Impairment of cognitive function can lead to difficulties with memory, attention, learning, coordination and sleep disturbances [12] and can persist for days, months or even years following the initial injury. Other long-term deficiencies include: language and communication problems (19%), dysarthria (30%), dysphagia (17%) [13], mood disorders [14,15] and cognitive impairment, even six months after mild TBI [16]. Another post-traumatic syndrome that can have a relatively delayed onset is post-traumatic epilepsy [14,15,17]. While all epilepsies are seizure disorders, not all seizures are epilepsy. As such, the incidence of early post-traumatic seizures (seizures immediately following, up to the first few days after the TBI) is higher than the incidence of post-traumatic epilepsy. Notably, about 25% of brain contusion patients and 32%–53% of patients with penetrating TBI develop different degrees of early post-traumatic seizures. Post-traumatic seizures also seem to be more prevalent following severe TBIs, although mild and moderate TBIs can also result in seizures [18]. Considering the negative impact of these numerous disorders associated with post-traumatic deficiencies, as well as the significant numbers of people suffering from the chronic effects of TBIs, research efforts are underway in the hopes of better understanding the pathogenic progression, and developing successful treatments of and diagnostic criterion for TBI. 

## 2. A Brief Review of Experimental TBI Animal Models

In view of the heterogeneous clinical nature of TBI, numerous animal models have been developed for experimentation. Although larger animals are closer in size and physiology to humans, rodents are a valuable and commonly-used model in TBI research. Their modest cost, biological similarities, more manageable size and standardized outcome measurements are all advantageous. Such models have been incorporated for studies aimed at improving our understanding of the detrimental, complex molecular cascades that are initiated by head trauma, as well as the long-term neurological and behavioral consequences. Therefore, unless otherwise indicated, this review focuses on data from animal models (primarily rodents) of TBI. Among them, several models are widely used in research: fluid percussion injury (FPI) [19,20,21,22], cortical impact injury (CCI) [23,24,25], penetrating ballistic-like brain injury [26], weight drop/impact acceleration injury [27] and blast TBI injury [28,29]. Although we will highlight the two most highly-cited animal models, FPI and CCI, it is important to note that many similarities with regard to the general neuroinflammatory responses are observed across rodent models of TBI, despite the different methods and modalities of the injuries.

### 2.1. Percussion Injury Animal Models

In percussive injury models, there are two devices that are most commonly incorporated into experiments. In the FPI model, the insult is inflicted by a pendulum striking the piston of a reservoir of fluid, and this generates a fluid pressure pulse that is delivered to the intact dura, via a syringe secured over an opened midline or lateral craniotomy [30,31]. In the second percussive TBI, an injury is delivered by a piston that is controlled either pneumatically or via a piezoelectric mechanism [32,33,34]. The percussion produces brief displacement and deformation of brain tissue, and the severity of injury depends on the strength of the pressure pulse [31], as well as the location of the craniotomy/injury. FPI models replicate clinical TBI without skull fracture [35] and, despite the craniotomy, are considered a closed head injury model [36]. The FPI model is often considered to be of mild to moderate severity, rather than severe [30]. A number of studies have shown that FPI can reliably reproduce intracranial hemorrhage [30,31], swelling [31,37], neuroinflammation [37,38,39] and gray matter damage [19,30], all of which are pathophysiological changes observed in human TBIs [40]. Based on the position of the craniotomy, FPI models can be divided into midline (centered on the sagittal suture), parasagittal (<3.5 mm lateral to midline) and lateral models (>3.5 mm lateral to midline; LFPI) [31,41,42,43]. The midline FPI model of TBI was first developed for use in cats and rabbits [37,44], secondly adapted for rats [30] and subsequently modified for use in mice [19,31]. FPI has also been used for studying TBI pathophysiology and pharmacology in other species [37,45,46], although the volume of literature pales in comparison to that for rodents.

In rodents, FPI produces a rapid combination of focal cortical contusion and diffuse subcortical (such as hippocampus and thalamus) neuronal injury. These can occur within minutes of the impact, progress to a loss of neurons by 12 h and are accompanied by a rapid neuroinflammatory response that is initially focused in the peri-injury region [38,39]. Neuronal death and neuroinflammatory signaling seem to peak at around three days after FPI and in many other models [38]. This inflammation persists, albeit at levels lower than the peak, well into chronic time points (≥1 month). In the days and months following the injury, progressive degenerative cascades that include chronic inflammation continue to be observed in a variety of brain regions implicated in higher cognitive functions. These include the hippocampus, thalamus, medial septum, striatum and amygdala [35,47,48]. It is often deduced that the neuropathology in these regions underlies the observed neurobehavioral and cognitive deficits that are commonly seen in the FPI model [36,49,50]. Of significance is the fact that analogous symptoms are often seen in patients with TBI-related injuries to corresponding brain regions [36,49].

### 2.2. Controlled Cortical Impact Injury Animal Model

The CCI model uses a pneumatic or electromagnetic impact device to drive a rigid impactor onto the exposed intact dura and mimics cortical tissue loss, acute subdural hematoma, axonal injury, concussion, blood-brain barrier (BBB) dysfunction and even coma [23,24,25]. It has been applied to a number of animals, such as ferrets [24], swine and monkeys [51] and, most prominently, rodents [23,25]. CCI is delivered to the intact dura through a craniotomy and results in deformation of the underlying cortex [23]. The damage created is highly reproducible and includes a rapid and sometimes widespread neuropathological damage. This damage is most prominent in the peri-injury area, includes neurodegenerative and neuroinflammatory responses [52], and can also encompass cortical, hippocampal and thalamic degeneration [53]. The histopathological severity of CCI rises with increasing cortical deformation, as does the cognitive impairment that is likely related to the extent of damage [54,55,56,57,58,59]. Similar to the FPI model, the neuropathology and associated cognitive and behavioral deficits after CCI persist chronically, and diffuse neuropathology is evident [60,61]. Similarly, the neuroinflammatory response appears to play a major role in both the early and chronic deficiencies observed following TBI. 

## 3. Mechanisms of Neuropathology Following TBI

It is now widely acknowledged that TBI is a complex multimodal disease process, not a single pathophysiological event [62]. It causes structural and functional damage, which lead to deficits resulting from both primary and secondary injury mechanisms [63]. The primary injury is the result of the immediate mechanical damage from direct contact and/or inertial forces to the brain that occurs at the moment of the traumatic impact. This damage can include direct neuronal, glial and other cellular damage, contusion, damage to blood vessels (hemorrhage) and axonal shearing [64,65]. Secondary injury evolves over minutes, to days, to months, to years after the primary injury and is the result of cascades of metabolic, cellular and molecular events. These occur concurrently with, and contribute to, alterations of endogenous neurochemical, inflammatory and neuroinflammatory mechanisms. Such mechanisms ultimately lead to brain cell death or rescue, plasticity, tissue damage and atrophy [35,66,67]. Many biochemical alterations responsible for secondary injury have also been identified. These include, perturbation of cellular calcium homeostasis, glutamate excitotoxicity, mitochondrial dysfunction, increased free radical generation, inflammation, neuroinflammation, increased lipid peroxidation, apoptosis and diffuse axonal injury (DAI) [68]. Interestingly, all of these alterations can be linked either directly or indirectly to neuroinflammation, and such inflammation has been implicated in the early and chronic components of TBI-induced neuropathology [69,70,71].

## 4. Inflammation Following TBI: An Immunological Perspective

### 4.1. Innate, Non-Specific Immune Response to TBI

At present, the prevailing viewpoint in the TBI field has been that most, if not all of the inflammation that follows a TBI can be considered components of the innate immune response [72,73,74]. However, accumulating evidence using updated technology suggests that specific adaptive immune mechanisms are also at play. Thus, a working operational definition is needed to define immune specificity after TBI, and few authors have adequately separated innate from adaptive immune components after a TBI. The early neuroinflammatory response across injuries and injury models occurs in a relatively stereotypical manner and can largely be considered to consist mainly of innate immune mechanisms. When damage to the brain takes place during TBI, it triggers the release and production of cytokines and chemokines, which activate receptors, and results in local and systemic immune responses [72,75,76]. The net effect of these innate inflammatory mediators is aimed at limiting the spread of the injury and restoring homeostatic balance [77].

### 4.2. Cytokines in TBI

Cytokines are categorized by structural and functional components, can be either pro- and/or anti-inflammatory and, in a classical immunological sense, are mediators of the cellular immune response, as well as of antibody synthesis and release. Cytokines can be synthesized and/or released by a wide variety of cells, including microglia, macrophages, T and B lymphocytes, endothelial and mast cells [78,79]. Although a full discussion of cytokine changes and functions after TBI is beyond the scope of this review, several reports indicate that interleukin (IL) IL1-β, IL18 and tumor necrosis factor alpha (TNFα) are involved in the onset and development of the inflammatory cascade after TBI in rodents and humans [72,73,74]. IL1-β binds to IL1-receptors, primarily localized on microglia and astrocytes in the brain, but also to other cell types, including infiltrating immune cells [80,81]. Activation of the neuroglial and immune cell IL1-receptors initiates the production and release of inflammatory cytokines, including increased production of IL1-β and IL18 [82]. This results in a self-perpetuating, pro-inflammatory environment, which may be damaging to the CNS parenchyma [75,76,83,84]. The damaging effects of IL1-β can also be related to activation of other pro-inflammatory pathways, such as, TNFα [85] and IL18 [72]. 

Several studies support the evidence of rapid and sustained induction of TNFα in damaged brain tissue, within one hour after TBI in rodent models [75,76,86]. TNFα triggers the production of other cytokines (IL1-β, IL6), chemokines [75] and nuclear factor kappa B (NF-κB) family (p50, p52 and p65) of transcription factors. Thus, TNFα is an important modulator of inflammation, at the transcriptional and translational levels, in the nervous system and in non-neural tissues [87,88]. IL18 also appears to play an important inflammatory role after TBI. IL18 has been shown to be elevated following a number of CNS inflammatory insults [89,90,91], including TBI [72]. In humans and rodents, the IL18 pathway can contribute to delayed neuronal injury, up to 14 days following TBI [72]. Activated neuroglial and immune cells in the area of injury secrete IL18, which binds to the IL18 receptor. Activation of the IL18 receptor initiates inflammatory signaling cascades [92]. Thus, cytokines, some of which have been mentioned here, are major contributors to the inflammatory and neuroinflammatory response. 

### 4.3. Chemokines in TBI

Chemokines (CCL) are chemotactic inflammatory proteins that mediate interactions among inflammatory cells and target cells. In general, chemokines are typically 10 KDa or smaller. CCLs are synthesized and/or released along with other mediator molecules by a variety of cell types that include: astrocytes, microglia, macrophages, eosinophils, neutrophils, dendritic cells, mast cells and natural killer cells (NK cells) [93,94,95]. Release of chemokines serves to chemotactically guide receptor-sensitive cells, primarily through activation of G protein-coupled receptors [96]. Similar to cytokines, chemokines can be either pro- and/or anti-inflammatory. After a TBI, chemokines contribute to the attraction of a wide range of immune cells to the site of damage [97,98]. The specific activities of classes of chemokines have been elucidated following TBIs [99,100,101]. Although a full discussion is beyond the scope of this review, one example, CXC chemokines, activates the migration of neutrophils to the site of the lesion [96]. Alternatively, chemokines CCL2, CCL3, CCL5, CCL7, CCL8, CCL13, CCL17 and CCL22 attract monocytes and macrophages [102,103,104]. Other chemokines, such as, CCL1, CCL2, CCL17 and CCL22, are involved in the recruitment of T-lymphocytes [103]. 

Another important role of cytokine and chemokine release is to activate pattern recognition receptors (PRRs). PRRs are proteins of the innate immune system and identify danger-associated molecular patterns (DAMPs) of cellular stress. This identification, and the ensuing response, helps defend against cell and/or tissue damage [105,106]. The PRRs are divided into several subgroups, depending on cell localization, type and function. One such group is the nucleotide-binding domain leucine-rich repeats (NLRs) [107], which are also called the nucleotide oligomerization domain (NOD)-like receptors. These receptors are located in the cytoplasm and help to regulate the host inflammatory, apoptotic and innate immune responses [108,109]. The NLR family of proteins can be activated by multiple types of cell/tissue damage that are seen in TBI and can form multi-protein complexes called “inflammasomes” [110]. The unique compositions of these inflammasomes depend on the extent and type of cell and tissue damage. Some reports suggested the specific contributions of NLR family proteins (NLRP3-inflammasome) after TBI [110,111]. The NLRP3-inflammasome has been detected in neurons, astrocytes and microglia in the cortex after TBI [110], and the NLRP3-inflammasome complex is associated with increases in the aforementioned IL1-β and IL18 [108,110,112]. Interestingly, the NLRP3-inflammasome has also been demonstrated to associate with other CNS inflammatory disorders, including Alzheimer’s disease (AD) [113], which is an increased risk factor following TBI. 

Thus, the overall contributions of the cytokines and chemokines released after TBI are mediated by the release, and subsequent recruitment of immune cells to the site of injury, and to coordinate the ensuing activity of these cells. These immune cells are an essential part of the innate immune response and also the putative transition to the adaptive immune response and will be discussed below. 

### 4.4. Cellular Immune Response to TBI

Several reports show that TBI and the associated neuroinflammation lead to local deposition of specific immune cells at the peri-injury area and beyond [97,114,115]. In addition, peripheral inflammation can also influence TBI outcomes [116]. There appears to be a rapid expansion and activation of peripheral immune cells after experimental TBI, as well as a significant extravasation of immune cells from the spleen, resulting in splenic hypotrophy following TBI [117,118]. It is also known that some of these immune cells that exit the spleen can contribute to the innate immune response, and some may also contribute to an ensuing adaptive immune response following a TBI [116,119,120]. Similarly, it is also known that some of these immune cells will infiltrate the CNS [121,122,123]. Immune cells involved in coordinating the innate immune response include: (1) monocytes, which develop into macrophages; (2) mast cells; (3) granulocytes (basophils, eosinophils and neutrophils); (4) dendritic cells (DC); and (5) natural killer cells.

Neutrophils and macrophages strongly infiltrate the brain in the early phase of TBI [121,123,124]. Neutrophils appear to be the most numerous type of granulocytes and possess high phagocytic potential. These cells are highly migratory and were reported to be involved in phagocytosing damaged elements within the brain parenchyma following a TBI [115,122]. The processes whereby neutrophils function are by: (1) secreting lysosomal enzymes; (2) releasing free radicals; (3) decreasing blood flow by direct physical microvascular occlusion; (4) increasing vascular permeability [125,126,127]. Interestingly, neutrophils can be found in the microvasculature lining the peri-injury region by as early as 2 h after injury and in brain parenchyma shortly thereafter [115,122]. Thus, neutrophils can contribute to the development of BBB breakdown and subsequent brain edema formation [122,128]. It is possible that neutrophils contribute to the secondary damage seen after TBI. Consistent with this notion, blocking neutrophil migration and adhesion decreases the total area of neuronal damage after a TBI in rabbits [129]. 

It has been hypothesized that accumulation of DCs at the site of damaged brain parenchyma can negatively contribute to brain tissue damage after the onset of TBI [122,130]. Infiltrating DCs may be activated by contact with the damaged cells at the site of injury. Antigen materials get processed by DCs, which can than travel to distant lymph nodes, present antigen and generate a local immune response. Once this response is initiated, antigen-specific T cells may migrate into CNS parenchyma and cause extended, chronic damage to the brain [131,132]. T cell accumulation at the site of injury, concurrent with DC accumulation, has also been indicated to negatively influence TBI outcomes [115].

Activated macrophages and/or microglia also contribute to neuronal damage [122,133]. It has been reported that different molecules released after TBI contribute to microglia/macrophage phenotype shifts [134,135]. Although the utility of classifying the M1/M2 phenotype in vivo has come into question [136,137], it is still useful to review the potential impact of the different phenotypes. For example, damaged endothelial cells can mediate microglia/macrophage polarization through secretion of cytokines [138], such as TNF-α, IL-6, IL-25, transforming growth factor beta (TGF-β), interferon-gamma (IFN-gamma), including substance-P and lipocalin-2 [139,140,141]. Additionally, infiltrating peripheral immune cells (T lymphocytes specifically) can induce macrophage/microglial phenotype transformation [142]. Recent studies suggest that such phenotype changes can alter neurogenesis after TBI, such that macrophage polarization from M2 toward M1 stimulates the release of soluble factors that impair basal neurogenesis [138], and this may impair functional recovery [134,143]. Thus, macrophages and/or microglial cells can be acted upon by a number of factors that ultimately determine the functional consequences of these cell types.

Macrophages, including both resident (microglia) and infiltrating (peripheral macrophages), are observed in relatively high numbers following TBIs [133]. Macrophages appeared to be abundant between 12 and 72 h and predominate in damaged cortical regions [122,144]. These cells accumulate near the area of injury [144], and this accumulation is a result of at least two mechanisms. One is through attractions by locally-secreted chemo- and cytokines released at the site of injury, as previously discussed. The other mechanism appears to occur via the T-cell activation. Once in the damaged area, the T cells get activated by direct contact with antigen presentation on DCs, macrophages and microglia [145,146]. It is this latter activation which is the hallmark of a transition from a non-specific innate immune response, to a specific adaptive immune response; e.g. T cell recognition of presented antigen. 

T lymphocytes play an important role in the development and maintenance of secondary brain injury after TBI, through engagement of different cell types and mechanisms. Steady increases in the number and composition of T cells at the site of injury are highly suggestive of a transition to an adaptive immune response following a TBI. The peak of brain tissue infiltration by T cells after TBI, seems to occur within a range of 1–5 days, although the data on this topic are inconsistent [147,148]. Despite these inconsistencies, it appears that gamma delta T cells (γδ T cells), which are a distinct class of T cells largely developed in thymus and express γδ receptors, rather than αβ T-cell receptor (TCR) on their surface, are early responders to the site of brain injury [149]. Combined with CD8+ T lymphocytes and CD4+ T-helper 1 (TH1) cells, these cells may worsen damage by cytotoxic and pro-inflammatory actions [150,151]. However, some suggest that the infiltration of immune cells into the CNS after TBI can also be neuroprotective [120,152,153]. This hypothesis is substantiated by the supposition that antigen-activated T cells provide protection, maintenance of neurological integrity and repair of tissue after a TBI [154]. Reports indicate that CD4+ T-helper 2 (TH2) cells specific for myelin basic protein (MBP) and CD4+ T cells may play such role of protection in neuronal survival [155,156]. Despite the pathogenic potential of anti-MBP T cells, they were also found in human immune system of healthy individuals [157,158]. It seems that regulatory T cells (CD4+ CD25+ Foxp3+) might also play important role in protection by suppressing autoimmune activity [159,160]. They derive from naive CD4 cells and are known for their immunosuppressive action, which downregulate the induction and proliferation of effector T cells [159,160].

Thus, it is possible that after TBI, the inflammatory sequelae result in antigen processing and presentation, as well as an eventual transition to an adaptive immune response. The implications of a transition to an adaptive immune response after a TBI are poorly understood and may have a positive and/or negative impact on the CNS. Here, we will summarize the existing data supporting an adaptive immune response after TBI, and we will provide a novel hypothesis to generate a foundation from which ensuing studies can occur in the context of adaptive immunity and TBI.

### 4.5. Adaptive Immune Response to TBI

Although scant, following TBI, some studies have provided strong evidence for a switch from a non-specific innate immune response, to a specific, adaptive immune response. As such, a paradigm shift in thinking may be necessary to fully understand and appreciate the sequelae that occur in the early and chronic stages after a TBI. A switch to an adaptive immune response has occurred, once antigen is processed and presented by professional antigen-presenting cells (APCs), and T cells recognize the presented antigen. The evidence supporting the transition to an adaptive immune response following TBI has been observed following retinal crush studies [161,162], fluid percussion injury in mice [116] and in human TBI patients [163]. The cellular components of the adaptive immune response may entail resident brain cells and/or infiltrating immune cells, as described above. In the case of infiltrating immune cells, they can gain access to the CNS via the compromised BBB, as well as the aforementioned chemotactic signaling. The humoral component of the adaptive immunity can be initially mediated by B cells and subsequently by T cells, which produce antigen-specific antibodies. Although the evidence is lacking for a direct connection between antibody production by T cells and neurodegeneration, accumulating evidence supports a role in the adaptive immune response in neurodegeneration. Once a transition from an adaptive immune response occurs, the possible outcomes have the potential to profoundly influence the outcomes for TBI. 

In the case of most TBIs, the injuries are non-penetrating; thus, it would seem, if an adaptive immune response is occurring, then the antibody response is likely to be against self-antigen. Indeed, in human patients, evidence for this is found in the fact that antibodies to glial fibrillary acidic protein (GFAP)-fragments have been observed in the cerebral spinal fluid (CSF), at various time points after a TBI [164]. Moreover, proteolytic fragments of MBP, neuron specific enolase and to ubiquitin D-terminal hydrolase-L-1 (UCH-L1), which is a highly specific protein to neurons and an essential component of the ubiquitin proteasome system, have also been observed in humans and animal models of TBI [165,166,167,168]. Moreover, auto-reactive T cell responses to MBP have been documented in humans after TBI [169], further supporting a switch to an adaptive immune response. Considering that white matter loss has been reported after TBI in humans and animals, the observation of antibodies and T cells to MBP could have a tremendous implication on the loss of white matter and axonal degeneration that is observed at chronic time points after a TBI. A potential consequence of antibody production and the implied transition to an adaptive immune response is that memory immune cells are formed. It is possible that reactivation of the memory T cells, by another injury or perhaps by other neuroinflammatory stimulus, such as bacterial or viral infections [170], might re-open the BBB and expose the memory immune cells to self-antigens, such as GFAP, UCH-L1 or MBP. In this scenario, the subsequent appearance of post-traumatic syndromes may only appear after appropriate spatial and temporal stimuli, hence explaining the wide variation in when, why and what types of post-traumatic syndromes appear. Therefore, the development of post-TBI auto-antibody response might be highly pathogenic and contribute to chronic neuropathology that persists for a relatively long duration (days, week, months, years) after injury, and there is evidence in support of this notion from a wide swath of neurological and immunological studies [171,172,173,174].

Resident brain immune cells, such as microglial cells, infiltrate the CNS very early during development [175,176] and do not seem as likely a candidate to present self-antigen, despite the fact that these resident microglial cells are highly competent, professional APCs, and they can also express major histocompatibility complex class II molecules (MHCII). Considering that resident microglial cells in the brain are continuously exposed to GFAP, UCH-L1 and MBP and there does not seem to be any auto-reactivity in normal conditions, one alternative scenario is that infiltrating immune cells are responding to the antigenic peptide fragments of GFAP, UCH-L1 and MBP [165,166,167,177,178,179,180].

Another important factor to consider in the context of an adaptive immune response after TBI is the recent reports of two distinct lymphatic portals that directly service the brain. First, sitting subjacent to the superior sagittal sinus is a lymphatic area that has recently been shown to have white blood cells that dip in and out of the CNS, even in normal conditions [181,182]. Immune signals from the CNS have been shown to directly signal through this lymphatic portal and to initiate a global immune response that can exacerbate the severity of an injury [147,183,184]. Second is the “glymphatic” system, which allows for clearance of CNS waste products and soluble proteins. This system also has been shown to provide a substrate for the signaling of CNS components to the more classically-defined peripheral lymphatic system [184,185,186,187]. In either of these two CNS “lymphatic compartments”, there can be a rapid communication between the CNS and the periphery, and this signaling can exacerbate injuries, such as stroke or TBI [184,188]. In the stroke literature, removing the spleen (splenectomy) and, therefore, reducing the number of B and T cells capable of responding to the TBI results in a significant improvement in lesion size and functional outcome measures. However, the data were inconsistent between humans and rodent models [189,190,191]; thus, the true functional implications require further examination. Interestingly, other studies that have blocked the expansion and activation of B and T cells in the spleen after a TBI have demonstrated significant neuroprotection [116]. Thus, the role of peripheral immune cells after TBI might provide novel targets for the development of therapeutic options to treat TBIs and post-traumatic syndromes.

## 5. Conclusions

TBI remains a complex, multi-system pathology, with a wide-ranging potential for short- and long-term detrimental outcomes. Using the existing and newly-developed animal models, as well as clinical and translational studies, research continues to unravel the complex interactions between the brain, the periphery and the immune system. New understanding of these interactions will lead to novel therapeutic targets, with the hope of improving the outcomes for millions of people each year.

## References

[1] Marr A.L., Coronado V.G. (2004). Central Nervous System Injury Surveillance Data Submission Standards—2002.

[2] Centers for Disease Control and Prevention (CDC) Traumatic Brain Injury in the United States: Fact Sheet. https://www.cdc.gov/traumaticbraininjury/get_the_facts.html.

[3] Faul M.X., Xu L., Wald M.M., Coronad V.G. Traumatic Brain Injury in the United States: Emergency Department Visits, Hospitalizations and Deaths 2002–2006. https://www.cdc.gov/traumaticbraininjury/pdf/blue_book.pdf.

[4] Zaloshnja E., Miller T., Langlois J.A., Selassie A.W. (2008). Prevalence of long-term disability from traumatic brain injury in the civilian population of the United States, 2005. J. Head Trauma Rehabil..

[5] Centers for Disease Control and Prevention (CDC), United States Department of Defense (DOD), VA Leadership Panel Report to Congress on Traumatic Brain Injury in the United States: Understanding the Public Health Problem among Current and Former Military Personnel. https://www.cdc.gov/traumaticbraininjury/pdf/report_to_congress_on_traumatic_brain_injury_2013-a.pdf.

[6] Teasdale G., Jennett B. (1974). Assessment of coma and impaired consciousness. A practical scale. Lancet.

[7] Jennett B., Bond M. (1975). Assessment of outcome after severe brain damage. Lancet.

[8] Nakase-Richardson R., Sherer M., Seel R.T., Hart T., Hanks R., Arango-Lasprilla J.C., Yablon S.A., Sander A.M., Barnett S.D., Walker W.C. (2011). Utility of post-traumatic amnesia in predicting 1-year productivity following traumatic brain injury: Comparison of the Russell and Mississippi PTA classification intervals. J. Neurol. Neurosurg. Psychiatry.

[9] Brenner L.A., Vanderploeg R.D., Terrio H. (2009). Assessment and diagnosis of mild traumatic brain injury, posttraumatic stress disorder, and other polytrauma conditions: Burden of adversity hypothesis. Rehabil. Psychol..

[10] Turan N., Miller B.A., Heider R.A., Nadeem M., Sayeed I., Stein D.G., Pradilla G. (2016). Neurobehavioral testing in subarachnoid hemorrhage: A review of methods and current findings in rodents. J. Cereb. Blood Flow Metab..

[11] Riggio S., Wong M. (2009). Neurobehavioral sequelae of traumatic brain injury. Mt. Sinai J. Med..

[12] Walker W.C., Pickett T.C. (2007). Motor impairment after severe traumatic brain injury: A longitudinal multicenter study. J. Rehabil. Res. Dev..

[13] Safaz I., Alaca R., Yasar E., Tok F., Yilmaz B. (2008). Medical complications, physical function and communication skills in patients with traumatic brain injury: A single centre 5-year experience. Brain Inj..

[14] Rosenthal M., Christensen B.K., Ross T.P. (1998). Depression following traumatic brain injury. Arch. Phys. Med. Rehabil..

[15] Hart T., Brenner L., Clark A.N., Bogner J.A., Novack T.A., Chervoneva I., Nakase-Richardson R., Arango-Lasprilla J.C. (2011). Major and minor depression after traumatic brain injury. Arch. Phys. Med. Rehabil..

[16] Stulemeijer M., Vos P.E., Bleijenberg G., van der Werf S.P. (2007). Cognitive complaints after mild traumatic brain injury: Things are not always what they seem. J. Psychosom. Res..

[17] Agrawal A., Timothy J., Pandit L., Manju M. (2006). Post-traumatic epilepsy: An overview. Clin. Neurol. Neurosurg..

[18] Bazarian J.J., Cernak I., Noble-Haeusslein L., Potolicchio S., Temkin N. (2009). Long-term neurologic outcomes after traumatic brain injury. J. Head Trauma Rehabil..

[19] Carbonell W.S., Maris D.O., McCall T., Grady M.S. (1998). Adaptation of the fluid percussion injury model to the mouse. J. Neurotrauma.

[20] Dixon C.E., Lighthall J.W., Anderson T.E. (1988). Physiologic, histopathologic, and cineradiographic characterization of a new fluid-percussion model of experimental brain injury in the rat. J. Neurotrauma.

[21] Dixon C.E., Lyeth B.G., Povlishock J.T., Findling R.L., Hamm R.J., Marmarou A., Young H.F., Hayes R.L. (1987). A fluid percussion model of experimental brain injury in the rat. J. Neurosurg..

[22] Mukherjee S., Zeitouni S., Cavarsan C.F., Shapiro L.A. (2013). Increased seizure susceptibility in mice 30 days after fluid percussion injury. Front. Neurol..

[23] Dixon C.E., Clifton G.L., Lighthall J.W., Yaghmai A.A., Hayes R.L. (1991). A controlled cortical impact model of traumatic brain injury in the rat. J. Neurosci. Methods.

[24] Lighthall J.W. (1988). Controlled cortical impact: A new experimental brain injury model. J. Neurotrauma.

[25] Smith D.H., Soares H.D., Pierce J.S., Perlman K.G., Saatman K.E., Meaney D.F., Dixon C.E., McIntosh T.K. (1995). A model of parasagittal controlled cortical impact in the mouse: Cognitive and histopathologic effects. J. Neurotrauma.

[26] Williams A.J., Hartings J.A., Lu X.C., Rolli M.L., Dave J.R., Tortella F.C. (2005). Characterization of a new rat model of penetrating ballistic brain injury. J. Neurotrauma.

[27] Marmarou A., Foda M.A., van den Brink W., Campbell J., Kita H., Demetriadou K. (1994). A new model of diffuse brain injury in rats. Part I: Pathophysiology and biomechanics. J. Neurosurg..

[28] Cernak I., Savic J., Malicevic Z., Zunic G., Radosevic P., Ivanovic I., Davidovic L. (1996). Involvement of the central nervous system in the general response to pulmonary blast injury. J. Trauma.

[29] Warden D. (2006). Military tbi during the iraq and afghanistan wars. J. Head Trauma Rehabil..

[30] McIntosh T.K., Noble L., Andrews B., Faden A.I. (1987). Traumatic brain injury in the rat: Characterization of a midline fluid-percussion model. Cent. Nerv. Syst. Trauma.

[31] McIntosh T.K., Vink R., Noble L., Yamakami I., Fernyak S., Soares H., Faden A.L. (1989). Traumatic brain injury in the rat: Characterization of a lateral fluid-percussion model. Neuroscience.

[32] Kabadi S.V., Hilton G.D., Stoica B.A., Zapple D.N., Faden A.I. (2010). Fluid-percussion-induced traumatic brain injury model in rats. Nat. Protoc..

[33] Walter B., Bauer R., Fritz H., Jochum T., Wunder L., Zwiener U. (1999). Evaluation of micro tip pressure transducers for the measurement of intracerebral pressure transients induced by fluid percussion. Exp. Toxicol. Pathol..

[34] Alder J., Fujioka W., Lifshitz J., Crockett D.P., Thakker-Varia S. (2011). Lateral fluid percussion: Model of traumatic brain injury in mice. J. Vis. Exp..

[35] Thompson H.J., Lifshitz J., Marklund N., Grady M.S., Graham D.I., Hovda D.A., McIntosh T.K. (2005). Lateral fluid percussion brain injury: A 15-year review and evaluation. J. Neurotrauma.

[36] Morales D.M., Marklund N., Lebold D., Thompson H.J., Pitkanen A., Maxwell W.L., Longhi L., Laurer H., Maegele M., Neugebauer E. (2005). Experimental models of traumatic brain injury: Do we really need to build a better mousetrap?. Neuroscience.

[37] Hartl R., Medary M., Ruge M., Arfors K.E., Ghajar J. (1997). Blood-brain barrier breakdown occurs early after traumatic brain injury and is not related to white blood cell adherence. Acta Neurochir. Suppl..

[38] Das M., Leonardo C.C., Rangooni S., Pennypacker K.R., Mohapatra S., Mohapatra S.S. (2011). Lateral fluid percussion injury of the brain induces CCL20 inflammatory chemokine expression in rats. J. Neuroinflamm..

[39] Xiong Y., Mahmood A., Chopp M. (2013). Animal models of traumatic brain injury. Nat. Rev. Neurosci..

[40] Graham D.I., McIntosh T.K., Maxwell W.L., Nicoll J.A. (2000). Recent advances in neurotrauma. J. Neuropathol. Exp. Neurol..

[41] Sanders M.J., Dietrich W.D., Green E.J. (1999). Cognitive function following traumatic brain injury: Effects of injury severity and recovery period in a parasagittal fluid-percussive injury model. J. Neurotrauma.

[42] Vink R., Mullins P.G., Temple M.D., Bao W., Faden A.I. (2001). Small shifts in craniotomy position in the lateral fluid percussion injury model are associated with differential lesion development. J. Neurotrauma.

[43] Floyd C.L., Golden K.M., Black R.T., Hamm R.J., Lyeth B.G. (2002). Craniectomy position affects morris water maze performance and hippocampal cell loss after parasagittal fluid percussion. J. Neurotrauma.

[44] Hayes R.L., Stalhammar D., Povlishock J.T., Allen A.M., Galinat B.J., Becker D.P., Stonnington H.H. (1987). A new model of concussive brain injury in the cat produced by extradural fluid volume loading: II. Physiological and neuropathological observations. Brain Inj..

[45] Millen J.E., Glauser F.L., Fairman R.P. (1985). A comparison of physiological responses to percussive brain trauma in dogs and sheep. J. Neurosurg..

[46] Pfenninger E.G., Reith A., Breitig D., Grunert A., Ahnefeld F.W. (1989). Early changes of intracranial pressure, perfusion pressure, and blood flow after acute head injury. Part 1: An experimental study of the underlying pathophysiology. J. Neurosurg..

[47] Hicks R., Soares H., Smith D., McIntosh T. (1996). Temporal and spatial characterization of neuronal injury following lateral fluid-percussion brain injury in the rat. Acta Neuropathol..

[48] Liu Y.R., Cardamone L., Hogan R.E., Gregoire M.C., Williams J.P., Hicks R.J., Binns D., Koe A., Jones N.C., Myers D.E. (2010). Progressive metabolic and structural cerebral perturbations after traumatic brain injury: An in vivo imaging study in the rat. J. Nucl. Med..

[49] Hamm R.J. (2001). Neurobehavioral assessment of outcome following traumatic brain injury in rats: An evaluation of selected measures. J. Neurotrauma.

[50] Pierce J.E., Smith D.H., Trojanowski J.Q., McIntosh T.K. (1998). Enduring cognitive, neurobehavioral and histopathological changes persist for up to one year following severe experimental brain injury in rats. Neuroscience.

[51] King C., Robinson T., Dixon C.E., Rao G.R., Larnard D., Nemoto C.E. (2010). Brain temperature profiles during epidural cooling with the chillerpad in a monkey model of traumatic brain injury. J. Neurotrauma.

[52] Acosta S.A., Tajiri N., Shinozuka K., Ishikawa H., Grimmig B., Diamond D.M., Sanberg P.R., Bickford P.C., Kaneko Y., Borlongan C.V. (2013). Long-term upregulation of inflammation and suppression of cell proliferation in the brain of adult rats exposed to traumatic brain injury using the controlled cortical impact model. PLoS ONE.

[53] Hall E.D., Sullivan P.G., Gibson T.R., Pavel K.M., Thompson B.M., Scheff S.W. (2005). Spatial and temporal characteristics of neurodegeneration after controlled cortical impact in mice: More than a focal brain injury. J. Neurotrauma.

[54] Goodman J.C., Cherian L., Bryan R.M., Robertson C.S. (1994). Lateral cortical impact injury in rats: Pathologic effects of varying cortical compression and impact velocity. J. Neurotrauma.

[55] Saatman K.E., Feeko K.J., Pape R.L., Raghupathi R. (2006). Differential behavioral and histopathological responses to graded cortical impact injury in mice. J. Neurotrauma.

[56] Petraglia A.L., Plog B.A., Dayawansa S., Chen M., Dashnaw M.L., Czerniecka K., Walker C.T., Viterise T., Hyrien O., Iliff J.J. (2014). The spectrum of neurobehavioral sequelae after repetitive mild traumatic brain injury: A novel mouse model of chronic traumatic encephalopathy. J. Neurotrauma.

[57] Fox G.B., Fan L., Levasseur R.A., Faden A.I. (1998). Sustained sensory/motor and cognitive deficits with neuronal apoptosis following controlled cortical impact brain injury in the mouse. J. Neurotrauma.

[58] Washington P.M., Forcelli P.A., Wilkins T., Zapple D.N., Parsadanian M., Burns M.P. (2012). The effect of injury severity on behavior: A phenotypic study of cognitive and emotional deficits after mild, moderate, and severe controlled cortical impact injury in mice. J. Neurotrauma.

[59] Marklund N., Hillered L. (2011). Animal modelling of traumatic brain injury in preclinical drug development: Where do we go from here?. Br. J. Pharmacol..

[60] Dixon C.E., Kraus M.F., Kline A.E., Ma X., Yan H.Q., Griffith R.G., Wolfson B.M., Marion D.W. (1999). Amantadine improves water maze performance without affecting motor behavior following traumatic brain injury in rats. Restor. Neurol. Neurosci..

[61] Dixon C.E., Kochanek P.M., Yan H.Q., Schiding J.K., Griffith R.G., Baum E., Marion D.W., DeKosky S.T. (1999). One-year study of spatial memory performance, brain morphology, and cholinergic markers after moderate controlled cortical impact in rats. J. Neurotrauma.

[62] Masel B.E., DeWitt D.S. (2010). Traumatic brain injury: A disease process, not an event. J. Neurotrauma.

[63] Davis A.E. (2000). Mechanisms of traumatic brain injury: Biomechanical, structural and cellular considerations. Crit. Care Nurs. Q..

[64] Gaetz M. (2004). The neurophysiology of brain injury. Clin. Neurophysiol..

[65] Cernak I. (2005). Animal models of head trauma. NeuroRx.

[66] Bramlett H.M., Dietrich W.D. (2007). Progressive damage after brain and spinal cord injury: Pathomechanisms and treatment strategies. Prog. Brain Res..

[67] Marklund N., Bakshi A., Castelbuono D.J., Conte V., McIntosh T.K. (2006). Evaluation of pharmacological treatment strategies in traumatic brain injury. Curr. Pharm. Des..

[68] Povlishock J.T., Christman C.W. (1995). The pathobiology of traumatically induced axonal injury in animals and humans: A review of current thoughts. J. Neurotrauma.

[69] Arvin B., Neville L.F., Barone F.C., Feuerstein G.Z. (1995). Brain injury and inflammation. A putative role of TNF alpha. Ann. N. Y. Acad. Sci..

[70] Isaksson J., Lewen A., Hillered L., Olsson Y. (1997). Up-regulation of intercellular adhesion molecule 1 in cerebral microvessels after cortical contusion trauma in a rat model. Acta Neuropathol..

[71] Yang K., Mu X.S., Xue J.J., Whitson J., Salminen A., dixon C.E., Liu P.K., Hayes R.L. (1994). Increased expression of c-fos mRNA and AP-1 transcription factors after cortical impact injury in rats. Brain Res..

[72] Yatsiv I., Morganti-Kossmann M.C., Perez D., Dinarello C.A., Novick D., Rubinstein M., Otto V.I., Rancan M., Kossmann T., Redaelli C.A. (2002). Elevated intracranial IL-18 in humans and mice after traumatic brain injury and evidence of neuroprotective effects of IL-18-binding protein after experimental closed head injury. J. Cereb. Blood Flow Metab..

[73] Hutchinson P.J., O’Connell M.T., Rothwell N.J., Hopkins S.J., Nortje J., Carpenter K.L., Timofeev I., Al-Rawi P.G., Menon D.K., Pickard J.D. (2007). Inflammation in human brain injury: Intracerebral concentrations of IL-1alpha, IL-1beta, and their endogenous inhibitor IL-1ra. J. Neurotrauma.

[74] Utagawa A., Truettner J.S., Dietrich W.D., Bramlett H.M. (2008). Systemic inflammation exacerbates behavioral and histopathological consequences of isolated traumatic brain injury in rats. Exp. Neurol..

[75] Minami M., Kuraishi Y., Satoh M. (1991). Effects of kainic acid on messenger RNA levels of IL-1 beta, IL-6, TNF alpha and lif in the rat brain. Biochem. Biophys. Res. Commun..

[76] Liu T., Clark R.K., McDonnell P.C., Young P.R., White R.F., Barone F.C., Feuerstein G.Z. (1994). Tumor necrosis factor-alpha expression in ischemic neurons. Stroke.

[77] Chizzolini C., Dayer J.M., Miossec P. (2009). Cytokines in chronic rheumatic diseases: Is everything lack of homeostatic balance?. Arthritis Res. Ther..

[78] Iwasaki A., Medzhitov R. (2010). Regulation of adaptive immunity by the innate immune system. Science.

[79] Zhang J.M., An J. (2007). Cytokines, inflammation, and pain. Int. Anesthesiol. Clin..

[80] Dinarello C.A. (2009). Immunological and inflammatory functions of the interleukin-1 family. Annu. Rev. Immunol..

[81] Garlanda C., Dinarello C.A., Mantovani A. (2013). The interleukin-1 family: Back to the future. Immunity.

[82] Pearson V.L., Rothwell N.J., Toulmond S. (1999). Excitotoxic brain damage in the rat induces interleukin-1beta protein in microglia and astrocytes: Correlation with the progression of cell death. Glia.

[83] Dinarello C.A. (2005). Blocking IL-1 in systemic inflammation. J. Exp. Med..

[84] Dinarello C.A. (2006). Interleukin 1 and interleukin 18 as mediators of inflammation and the aging process. Am. J. Clin. Nutr..

[85] Lu K.T., Wang Y.W., Wo Y.Y., Yang Y.L. (2005). Extracellular signal-regulated kinase-mediated IL-1-induced cortical neuron damage during traumatic brain injury. Neurosci. Lett..

[86] Liu T., Young P.R., McDonnell P.C., White R.F., Barone F.C., Feuerstein G.Z. (1993). Cytokine-induced neutrophil chemoattractant mRNA expressed in cerebral ischemia. Neurosci. Lett..

[87] Grilli M., Memo M. (1999). Nuclear factor-kappaB/Rel proteins: A point of convergence of signalling pathways relevant in neuronal function and dysfunction. Biochem. Pharmacol..

[88] Baeuerle P.A., Baltimore D. (1996). NF-kappa B: Ten years after. Cell.

[89] Jander S., Stoll G. (1998). Differential induction of interleukin-12, interleukin-18, and interleukin-1beta converting enzyme mRNA in experimental autoimmune encephalomyelitis of the lewis rat. J. Neuroimmunol..

[90] Losy J., Niezgoda A. (2001). IL-18 in patients with multiple sclerosis. Acta Neurol. Scand..

[91] Fassbender K., Mielke O., Bertsch T., Muehlhauser F., Hennerici M., Kurimoto M., Rossol S. (1999). Interferon-gamma-inducing factor (IL-18) and interferon-gamma in inflammatory CNS diseases. Neurology.

[92] Sims J.E., Smith D.E. (2010). The IL-1 family: Regulators of immunity. Nat. Rev. Immunol..

[93] Kremlev S.G., Roberts R.L., Palmer C. (2004). Differential expression of chemokines and chemokine receptors during microglial activation and inhibition. J. Neuroimmunol..

[94] Gyoneva S., Ransohoff R.M. (2015). Inflammatory reaction after traumatic brain injury: Therapeutic potential of targeting cell-cell communication by chemokines. Trends Pharmacol. Sci..

[95] Choi S.S., Lee H.J., Lim I., Satoh J., Kim S.U. (2014). Human astrocytes: Secretome profiles of cytokines and chemokines. PLoS ONE.

[96] Ono S.J., Nakamura T., Miyazaki D., Ohbayashi M., Dawson M., Toda M. (2003). Chemokines: Roles in leukocyte development, trafficking, and effector function. J. Allergy Clin. Immunol..

[97] Helmy A., Carpenter K.L., Menon D.K., Pickard J.D., Hutchinson P.J. (2011). The cytokine response to human traumatic brain injury: Temporal profiles and evidence for cerebral parenchymal production. J. Cereb. Blood Flow Metab..

[98] Helmy A., Antoniades C.A., Guilfoyle M.R., Carpenter K.L., Hutchinson P.J. (2012). Principal component analysis of the cytokine and chemokine response to human traumatic brain injury. PLoS ONE.

[99] Glabinski A.R., Tani M., Aras S., Stoler M.H., Tuohy V.K., Ransohoff R.M. (1995). Regulation and function of central nervous system chemokines. Int. J. Dev. Neurosci..

[100] Ghirnikar R.S., Lee Y.L., Eng L.F. (1998). Inflammation in traumatic brain injury: Role of cytokines and chemokines. Neurochem. Res..

[101] Cartier L., Hartley O., Dubois-Dauphin M., Krause K.H. (2005). Chemokine receptors in the central nervous system: Role in brain inflammation and neurodegenerative diseases. Brain Res. Brain Res. Rev..

[102] Proudfoot A.E., Handel T.M., Johnson Z., Lau E.K., LiWang P., Clark-Lewis I., Borlat F., Wells T.N., Kosco-Vilbois M.H. (2003). Glycosaminoglycan binding and oligomerization are essential for the in vivo activity of certain chemokines. Proc. Natl. Acad. Sci. USA.

[103] Mantovani A., Sica A., Sozzani S., Allavena P., Vecchi A., Locati M. (2004). The chemokine system in diverse forms of macrophage activation and polarization. Trends Immunol..

[104] Shi C., Pamer E.G. (2011). Monocyte recruitment during infection and inflammation. Nat. Rev. Immunol..

[105] Tang D., Kang R., Coyne C.B., Zeh H.J., Lotze M.T. (2012). PAMPs and DAMPs: Signal 0s that spur autophagy and immunity. Immunol. Rev..

[106] Sansonetti P.J. (2006). The innate signaling of dangers and the dangers of innate signaling. Nat. Immunol..

[107] Trinchieri G., Sher A. (2007). Cooperation of toll-like receptor signals in innate immune defence. Nat. Rev. Immunol..

[108] Ting J.P., Lovering R.C., Alnemri E.S., Bertin J., Boss J.M., Davis B.K., Flavell R.A., Girardin S.E., Godzik A., Harton J.A. (2008). The NLR gene family: A standard nomenclature. Immunity.

[109] Strober W., Murray P.J., Kitani A., Watanabe T. (2006). Signalling pathways and molecular interactions of NOD1 and NOD2. Nat. Rev. Immunol..

[110] Liu H.D., Li W., Chen Z.R., Hu Y.C., Zhang D.D., Shen W., Zhou M.L., Zhu L., Hang C.H. (2013). Expression of the NLRP3 inflammasome in cerebral cortex after traumatic brain injury in a rat model. Neurochem. Res..

[111] Needham E., Zandi M.S. (2014). Recent advances in the neuroimmunology of cell-surface CNS autoantibody syndromes, Alzheimer’s disease, traumatic brain injury and schizophrenia. J. Neurol..

[112] Martinon F., Burns K., Tschopp J. (2002). The inflammasome: A molecular platform triggering activation of inflammatory caspases and processing of proil-beta. Mol. Cell.

[113] Halle A., Hornung V., Petzold G.C., Stewart C.R., Monks B.G., Reinheckel T., Fitzgerald K.A., Latz E., Moore K.J., Golenbock D.T. (2008). The NALP3 inflammasome is involved in the innate immune response to amyloid-beta. Nat. Immunol..

[114] Trahanas D.M., Cuda C.M., Perlman H., Schwulst S.J. (2015). Differential activation of infiltrating monocyte-derived cells after mild and severe traumatic brain injury. Shock.

[115] Rhodes J. (2011). Peripheral immune cells in the pathology of traumatic brain injury?. Curr. Opin. Crit. Care.

[116] Tobin R.P., Mukherjee S., Kain J.M., Rogers S.K., Henderson S.K., Motal H.L., Newell Rogers M.K., Shapiro L.A. (2014). Traumatic brain injury causes selective, CD74-dependent peripheral lymphocyte activation that exacerbates neurodegeneration. Acta Neuropathol. Commun..

[117] Schwulst S.J., Trahanas D.M., Saber R., Perlman H. (2013). Traumatic brain injury-induced alterations in peripheral immunity. J. Trauma Acute Care Surg..

[118] Rasouli J., Lekhraj R., Ozbalik M., Lalezari P., Casper D. (2011). Brain-spleen inflammatory coupling: A literature review. Einstein J. Biol. Med..

[119] Schwartz M., Deczkowska A. (2016). Neurological disease as a failure of brain-immune crosstalk: The multiple faces of neuroinflammation. Trends Immunol..

[120] Schwartz M. (2003). Helping the body to cure itself: Immune modulation by therapeutic vaccination for spinal cord injury. J. Spinal Cord Med..

[121] Foley L.M., Hitchens T.K., Ho C., Janesko-Feldman K.L., Melick J.A., Bayir H., Kochanek P.M. (2009). Magnetic resonance imaging assessment of macrophage accumulation in mouse brain after experimental traumatic brain injury. J. Neurotrauma.

[122] Soares H.D., Hicks R.R., Smith D., McIntosh T.K. (1995). Inflammatory leukocytic recruitment and diffuse neuronal degeneration are separate pathological processes resulting from traumatic brain injury. J. Neurosci..

[123] Kenne E., Erlandsson A., Lindbom L., Hillered L., Clausen F. (2012). Neutrophil depletion reduces edema formation and tissue loss following traumatic brain injury in mice. J. Neuroinflamm..

[124] Clark R.S., Schiding J.K., Kaczorowski S.L., Marion D.W., Kochanek P.M. (1994). Neutrophil accumulation after traumatic brain injury in rats: Comparison of weight drop and controlled cortical impact models. J. Neurotrauma.

[125] Harlan J.M. (1985). Leukocyte-endothelial interactions. Blood.

[126] Kochanek P.M., Hallenbeck J.M. (1992). Polymorphonuclear leukocytes and monocytes/macrophages in the pathogenesis of cerebral ischemia and stroke. Stroke.

[127] Lucchesi B.R., Mullane K.M. (1986). Leukocytes and ischemia-induced myocardial injury. Annu. Rev. Pharmacol. Toxicol..

[128] Burke-Gaffney A., Keenan A.K. (1993). Modulation of human endothelial cell permeability by combinations of the cytokines interleukin-1 alpha/beta, tumor necrosis factor-alpha and interferon-gamma. Immunopharmacology.

[129] Clark W.M., Madden K.P., Rothlein R., Zivin J.A. (1991). Reduction of central nervous system ischemic injury in rabbits using leukocyte adhesion antibody treatment. Stroke.

[130] Schwartz M., Yoles E. (2005). Macrophages and dendritic cells treatment of spinal cord injury: From the bench to the clinic. Acta Neurochir. Suppl..

[131] Zindler E., Zipp F. (2010). Neuronal injury in chronic CNS inflammation. Best Pract. Res. Clin. Anaesthesiol..

[132] Herz J., Zipp F., Siffrin V. (2010). Neurodegeneration in autoimmune CNS inflammation. Exp. Neurol..

[133] Jin X., Ishii H., Bai Z., Itokazu T., Yamashita T. (2012). Temporal changes in cell marker expression and cellular infiltration in a controlled cortical impact model in adult male C57BL/6 mice. PLoS ONE.

[134] Hu X., Li P., Guo Y., Wang H., Leak R.K., Chen S., Gao Y., Chen J. (2012). Microglia/macrophage polarization dynamics reveal novel mechanism of injury expansion after focal cerebral ischemia. Stroke.

[135] Rolls A., Shechter R., London A., Segev Y., Jacob-Hirsch J., Amariglio N., Rechavi G., Schwartz M. (2008). Two faces of chondroitin sulfate proteoglycan in spinal cord repair: A role in microglia/macrophage activation. PLoS Med..

[136] Heppner F.L., Ransohoff R.M., Becher B. (2015). Immune attack: The role of inflammation in alzheimer disease. Nat. Rev. Neurosci..

[137] Martinez F.O., Gordon S. (2014). The M1 and M2 paradigm of macrophage activation: Time for reassessment. F1000Prime Rep..

[138] Verma S., Nakaoke R., Dohgu S., Banks W.A. (2006). Release of cytokines by brain endothelial cells: A polarized response to lipopolysaccharide. Brain Behav. Immun..

[139] Jang E., Lee S., Kim J.H., Kim J.H., Seo J.W., Lee W.H., Mori K., Nakao K., Suk K. (2013). Secreted protein lipocalin-2 promotes microglial M1 polarization. FASEB J..

[140] Starossom S.C., Mascanfroni I.D., Imitola J., Cao L., Raddassi K., Hernandez S.F., Bassil R., Croci D.O., Cerliani J.P., Delacour D. (2012). Galectin-1 deactivates classically activated microglia and protects from inflammation-induced neurodegeneration. Immunity.

[141] Rocher C., Singla D.K. (2013). SMAD-PI3K-Akt-mTOR pathway mediates BMP-7 polarization of monocytes into M2 macrophages. PLoS ONE.

[142] Biswas S.K., Mantovani A. (2010). Macrophage plasticity and interaction with lymphocyte subsets: Cancer as a paradigm. Nat. Immunol..

[143] Roughton K., Andreasson U., Blomgren K., Kalm M. (2013). Lipopolysaccharide-induced inflammation aggravates irradiation-induced injury to the young mouse brain. Dev. Neurosci..

[144] Mukherjee S., Katki K., Arisi G.M., Foresti M.L., Shapiro L.A. (2011). Early tbi-induced cytokine alterations are similarly detected by two distinct methods of multiplex assay. Front. Mol. Neurosci..

[145] Ni K., O’Neill H.C. (1997). The role of dendritic cells in T cell activation. Immunol. Cell Biol..

[146] Pozzi L.A., Maciaszek J.W., Rock K.L. (2005). Both dendritic cells and macrophages can stimulate naive CD8 T cells in vivo to proliferate, develop effector function, and differentiate into memory cells. J. Immunol..

[147] Kelso M.L., Gendelman H.E. (2014). Bridge between neuroimmunity and traumatic brain injury. Curr. Pharm. Des..

[148] Gyoneva S., Kim D., Katsumoto A., Kokiko-Cochran O.N., Lamb B.T., Ransohoff R.M. (2015). *Ccr2* deletion dissociates cavity size and tau pathology after mild traumatic brain injury. J. Neuroinflamm..

[149] Gelderblom M., Arunachalam P., Magnus T. (2014). Gammadelta T cells as early sensors of tissue damage and mediators of secondary neurodegeneration. Front. Cell Neurosci..

[150] Sobottka B., Harrer M.D., Ziegler U., Fischer K., Wiendl H., Hunig T., Becher B., Goebels N. (2009). Collateral bystander damage by myelin-directed CD8+ T cells causes axonal loss. Am. J. Pathol..

[151] Melzer N., Meuth S.G., Wiendl H. (2009). CD8+ T cells and neuronal damage: Direct and collateral mechanisms of cytotoxicity and impaired electrical excitability. FASEB J..

[152] Serpe C.J., Coers S., Sanders V.M., Jones K.J. (2003). CD4+ T, but not CD8+ or B, lymphocytes mediate facial motoneuron survival after facial nerve transection. Brain Behav. Immun..

[153] Schwartz M., Shechter R. (2010). Systemic inflammatory cells fight off neurodegenerative disease. Nat. Rev. Neurol..

[154] Cohen I.R. (1992). The cognitive paradigm and the immunological homunculus. Immunol. Today.

[155] Moalem G., Leibowitz-Amit R., Yoles E., Mor F., Cohen I.R., Schwartz M. (1999). Autoimmune T cells protect neurons from secondary degeneration after central nervous system axotomy. Nat. Med..

[156] Bradl M., Bauer J., Flugel A., Wekerle H., Lassmann H. (2005). Complementary contribution of CD4 and CD8 T lymphocytes to T-cell infiltration of the intact and the degenerative spinal cord. Am. J. Pathol..

[157] Burns J., Rosenzweig A., Zweiman B., Lisak R.P. (1983). Isolation of myelin basic protein-reactive T-cell lines from normal human blood. Cell. Immunol..

[158] Martin R., Jaraquemada D., Flerlage M., Richert J., Whitaker J., Long E.O., McFarlin D.E., McFarland H.F. (1990). Fine specificity and HLA restriction of myelin basic protein-specific cytotoxic T cell lines from multiple sclerosis patients and healthy individuals. J. Immunol..

[159] Sakaguchi S. (2005). Naturally arising Foxp3-expressing CD25^+^CD4^+^ regulatory T cells in immunological tolerance to self and non-self. Nat. Immunol..

[160] Fontenot J.D., Gavin M.A., Rudensky A.Y. (2003). Foxp3 programs the development and function of CD4+CD25+ regulatory T cells. Nat. Immunol..

[161] Ben Simon G.J., Hovda D.A., Harris N.G., Gomez-Pinilla F., Goldberg R.A. (2006). Traumatic brain injury induced neuroprotection of retinal ganglion cells to optic nerve crush. J. Neurotrauma.

[162] Fisher J., Levkovitch-Verbin H., Schori H., Yoles E., Butovsky O., Kaye J.F., Ben-Nun A., Schwartz M. (2001). Vaccination for neuroprotection in the mouse optic nerve: Implications for optic neuropathies. J. Neurosci..

[163] Hazeldine J., Lord J.M., Belli A. (2015). Traumatic brain injury and peripheral immune suppression: Primer and prospectus. Front. Neurol..

[164] Jesse S., Steinacker P., Cepek L., von Arnim C.A., Tumani H., Lehnert S., Kretzschmar H.A., Baier M., Otto M. (2009). Glial fibrillary acidic protein and protein s-100b: Different concentration pattern of glial proteins in cerebrospinal fluid of patients with alzheimer's disease and creutzfeldt-jakob disease. J. Alzheimers Dis..

[165] Posti J.P., Takala R.S., Runtti H., Newcombe V.F., Outtrim J., Katila A.J., Frantzen J., Ala-Seppala H., Coles J.P., Hossain M.I. (2016). The levels of glial fibrillary acidic protein and ubiquitin c-terminal hydrolase-l1 during the first week after a traumatic brain injury: Correlations with clinical and imaging findings. Neurosurgery.

[166] Mondello S., Kobeissy F., Vestri A., Hayes R.L., Kochanek P.M., Berger R.P. (2016). Serum concentrations of ubiquitin c-terminal hydrolase-l1 and glial fibrillary acidic protein after pediatric traumatic brain injury. Sci. Rep..

[167] Yan E.B., Satgunaseelan L., Paul E., Bye N., Nguyen P., Agyapomaa D., Kossmann T., Rosenfeld J.V., Morganti-Kossmann M.C. (2014). Post-traumatic hypoxia is associated with prolonged cerebral cytokine production, higher serum biomarker levels, and poor outcome in patients with severe traumatic brain injury. J. Neurotrauma.

[168] Ottens A.K., Golden E.C., Bustamante L., Hayes R.L., Denslow N.D., Wang K.K. (2008). Proteolysis of multiple myelin basic protein isoforms after neurotrauma: Characterization by mass spectrometry. J. Neurochem..

[169] Cox A.L., Coles A.J., Nortje J., Bradley P.G., Chatfield D.A., Thompson S.J., Menon D.K. (2006). An investigation of auto-reactivity after head injury. J. Neuroimmunol..

[170] Kalia V., Sarkar S., Ahmed R. (2010). Cd8 t-cell memory differentiation during acute and chronic viral infections. Adv. Exp. Med. Biol..

[171] Goverman J. (2009). Autoimmune t cell responses in the central nervous system. Nat. Rev. Immunol..

[172] McFarland H.F., Martin R. (2007). Multiple sclerosis: A complicated picture of autoimmunity. Nat. Immunol..

[173] Lucchinetti C., Bruck W., Parisi J., Scheithauer B., Rodriguez M., Lassmann H. (2000). Heterogeneity of multiple sclerosis lesions: Implications for the pathogenesis of demyelination. Ann. Neurol..

[174] Ransohoff R.M., Kivisakk P., Kidd G. (2003). Three or more routes for leukocyte migration into the central nervous system. Nat. Rev. Immunol..

[175] Ginhoux F., Lim S., Hoeffel G., Low D., Huber T. (2013). Origin and differentiation of microglia. Front. Cell Neurosci..

[176] Harry G.J. (2013). Microglia during development and aging. Pharmacol. Ther..

[177] Papa L., Brophy G.M., Welch R.D., Lewis L.M., Braga C.F., Tan C.N., Ameli N.J., Lopez M.A., Haeussler C.A., Mendez Giordano D.I. (2016). Time course and diagnostic accuracy of glial and neuronal blood biomarkers gfap and uch-l1 in a large cohort of trauma patients with and without mild traumatic brain injury. JAMA Neurol..

[178] Zoltewicz J.S., Scharf D., Yang B., Chawla A., Newsom K.J., Fang L. (2012). Characterization of antibodies that detect human gfap after traumatic brain injury. Biomark. Insights.

[179] Bogoslovsky T., Wilson D., Chen Y., Hanlon D., Gill J., Jeromin A., Song L., Moore C., Gong Y., Kenney K. (2017). Increases of plasma levels of glial fibrillary acidic protein, tau, and amyloid beta up to 90 days after traumatic brain injury. J. Neurotrauma.

[180] Su E., Bell M.J., Kochanek P.M., Wisniewski S.R., Bayir H., Clark R.S., Adelson P.D., Tyler-Kabara E.C., Janesko-Feldman K.L., Berger R.P. (2012). Increased csf concentrations of myelin basic protein after tbi in infants and children: Absence of significant effect of therapeutic hypothermia. Neurocrit. Care.

[181] Raper D., Louveau A., Kipnis J. (2016). How do meningeal lymphatic vessels drain the cns?. Trends Neurosci..

[182] Louveau A., Da Mesquita S., Kipnis J. (2016). Lymphatics in neurological disorders: A neuro-lympho-vascular component of multiple sclerosis and alzheimer's disease?. Neuron.

[183] Brait V.H., Arumugam T.V., Drummond G.R., Sobey C.G. (2012). Importance of t lymphocytes in brain injury, immunodeficiency, and recovery after cerebral ischemia. J. Cereb. Blood Flow Metab..

[184] Louveau A., Smirnov I., Keyes T.J., Eccles J.D., Rouhani S.J., Peske J.D., Derecki N.C., Castle D., Mandell J.W., Lee K.S. (2015). Structural and functional features of central nervous system lymphatic vessels. Nature.

[185] Yang L., Kress B.T., Weber H.J., Thiyagarajan M., Wang B., Deane R., Benveniste H., Iliff J.J., Nedergaard M. (2013). Evaluating glymphatic pathway function utilizing clinically relevant intrathecal infusion of csf tracer. J. Transl. Med..

[186] Iliff J.J., Lee H., Yu M., Feng T., Logan J., Nedergaard M., Benveniste H. (2013). Brain-wide pathway for waste clearance captured by contrast-enhanced mri. J. Clin. Invest..

[187] Xie L., Kang H., Xu Q., Chen M.J., Liao Y., Thiyagarajan M., O'Donnell J., Christensen D.J., Nicholson C., Iliff J.J. (2013). Sleep drives metabolite clearance from the adult brain. Science.

[188] Aspelund A., Antila S., Proulx S.T., Karlsen T.V., Karaman S., Detmar M., Wiig H., Alitalo K. (2015). A dural lymphatic vascular system that drains brain interstitial fluid and macromolecules. J. Exp. Med..

[189] Li M., Li F., Luo C., Shan Y., Zhang L., Qian Z., Zhu G., Lin J., Feng H. (2011). Immediate splenectomy decreases mortality and improves cognitive function of rats after severe traumatic brain injury. J. Trauma.

[190] Chu W., Li M., Li F., Hu R., Chen Z., Lin J., Feng H. (2013). Immediate splenectomy down-regulates the mapk-nf-kappab signaling pathway in rat brain after severe traumatic brain injury. J. Trauma Acute Care Surg..

[191] Teixeira P.G., Karamanos E., Okoye O.T., Talving P., Inaba K., Lam L., Demetriades D. (2013). Splenectomy in patients with traumatic brain injury: Protective or harmful? A national trauma data bank analysis. J. Trauma Acute Care Surg..

